# The role of primary tumor resection in colorectal cancer patients with asymptomatic, synchronous unresectable metastasis: Study protocol for a randomized controlled trial

**DOI:** 10.1186/s13063-016-1164-0

**Published:** 2016-01-19

**Authors:** Chang Woo Kim, Jeong-Heum Baek, Gyu-Seog Choi, Chang Sik Yu, Sung Bum Kang, Won Cheol Park, Bong Hwa Lee, Hyeong Rok Kim, Jae Hwan Oh, Jae-Hwang Kim, Seung-Yong Jeong, Jung Bae Ahn, Seung Hyuk Baik

**Affiliations:** Section of Colon and Rectal Surgery, Department of Surgery, Severance Hospital, Yonsei University College of Medicine, Seoul, Republic of Korea; Section of Colon and Rectal Surgery, Department of Surgery, Gachon University Gil Hospital, Incheon, Republic of Korea; Section of Colon and Rectal Surgery, Department of Surgery, Kyungpook National University Hospital, Daegu, Republic of Korea; Section of Colon and Rectal Surgery, Department of Surgery, Asan Medical Center, Seoul, Republic of Korea; Section of Colon and Rectal Surgery, Department of Surgery, Seoul National University Bundang Hospital, Bundang, Republic of Korea; Section of Colon and Rectal Surgery, Department of Surgery, Wonkwang University Hospital, Iksan, Republic of Korea; Section of Colon and Rectal Surgery, Department of Surgery, Hallym University Sacred Heart Hospital, Anyang, Republic of Korea; Section of Colon and Rectal Surgery, Department of Surgery, Chonnam National University Hospital, Gwangju, Republic of Korea; Center for Colorectal Cancer, National Cancer Center, Ilsan, Republic of Korea; Section of Colon and Rectal Surgery, Department of Surgery, Yeungnam University Hospital, Daegu, Republic of Korea; Section of Colon and Rectal Surgery, Department of Surgery, Seoul National University Hospital, Seoul, Republic of Korea; Department of Medical Oncology, Severance Hospital, Yonsei University College of Medicine, Seoul, Republic of Korea; Section of Colon and Rectal Surgery, Department of Surgery, Gangnam Severance Hospital, Yonsei University College of Medicine, 211 Eonju-ro, Gangnam-gu, Seoul, 135-720 Republic of Korea

**Keywords:** Chemotherapy, Colorectal cancer, Overall survival, Quality of life, Primary tumor resection

## Abstract

**Background:**

Approximately 20 % of all patients with colorectal cancer are diagnosed as having Stage IV cancer; 80 % of these present with unresectable metastatic lesions. It is controversial whether chemotherapy with or without primary tumor resection (PTR) is effective for the treatment of patients with colorectal cancer with unresectable metastasis. Primary tumor resection could prevent tumor-related complications such as intestinal obstruction, perforation, bleeding, or fistula. Moreover, it may be associated with an increase in overall survival. However, surgery delays the use of systemic chemotherapy and affects the systemic spread of malignancy.

**Methods/design:**

Patients with colon and upper rectal cancer patients with asymptomatic, synchronous, unresectable metastasis will be included after screening. They will be randomized and assigned to receive chemotherapy with or without PTR. The primary endpoint measure is 2-year overall survival rate and the secondary endpoint measures are primary tumor-related complications, quality of life, surgery-related morbidity and mortality, interventions with curative intent, chemotherapy-related toxicity, and total cost until death or study closing day. The authors hypothesize that the group receiving PTR following chemotherapy would show a 10 % improvement in 2-year overall survival, compared with the group receiving chemotherapy alone. The accrual period is 3 years and the follow-up period is 2 years. Based on the inequality design, a two-sided log-rank test with α-error of 0.05 and a power of 80 % was conducted. Allowing for a drop-out rate of 10 %, 480 patients (240 per group) will need to be recruited. Patients will be followed up at every 3 months for 3 years and then every 6 months for 2 years after the last patient has been randomized.

**Discussion:**

This randomized controlled trial aims to investigate whether PTR with chemotherapy shows better overall survival than chemotherapy alone for patients with asymptomatic, synchronous unresectable metastasis. This trial is expected to provide evidence so support clear treatment guidelines for patients with colorectal cancer with asymptomatic, synchronous unresectable metastasis.

**Trial registration:**

Clinicaltrials.gov NCT01978249.

## Background

Colorectal cancer is the third most common malignancy in the world. About 940,000 cases of colorectal cancer develop annually, and about 500,000 patients die annually [1]. Approximately 20 % of all patients with colorectal cancer are diagnosed as having Stage IV cancer; 80 % of these present with unresectable metastatic lesions [2].

The treatment of choice for colorectal cancer is radical surgery, and additional chemotherapy or radiotherapy is available to prevent residual microcarcinoma, distant metastasis, and recurrence. However, in this incurable situation, the treatment of choice is unclear. According to the National Comprehensive Cancer Network guidelines [3], chemotherapy for colorectal cancer with unresectable metastasis is recommended following palliative resection of primary tumor only if the patient shows tumor-related symptoms. When the patient has no tumor-related symptoms, the first treatment option is chemotherapy. Thereafter, surgery for the primary tumor and metastatic lesions is recommended if the metastatic lesions become resectable.

Primary tumor resection (PTR) requires time for the patient to recover, which subsequently delays systemic chemotherapy and affects the systemic spread of malignancy. Several studies showed no significant difference in overall survival rate between groups receiving PTR and groups not receiving PTR [4, 5]. Moreover, since the introduction of new chemotherapeutic agents and regimens, several phase III trials have revealed prolonged median overall survivals of 17–23 months for colorectal cancer patients with distant metastases [6–8]. Thus, some physicians prefer chemotherapy to PTR because of these drawbacks of PTR and because of the low tumor-related complication rates obtained with recently developed chemotherapeutic agents [4, 5, 9–11].

Conversely, many studies reported that PTR is necessary in patients with unresectable distant metastases [12–19]. Primary tumor resection could prevent tumor-related complications, such as intestinal obstruction, perforation, bleeding, or fistula [15, 17]. These complications are associated with poor oncologic outcomes as well as perioperative morbidity and mortality. Moreover, recent papers have reported significantly better overall survival rates for patients undergoing PTR than patients who do not have this treatment [12–14, 16, 18, 19]. We found similar results from data obtained at our institution [20]. Moreover, National Comprehensive Cancer Network guidelines for this issue are based on lower-level evidence (category 2A).

However, previous studies support each opposing conclusion, and these studies are all retrospective. Therefore, we cannot know whether PTR should be performed or not for the patients with unresectable distant metastasis. A prospective, randomized controlled trial is required for this reason. Thus, we have designed a clinical trial. The purpose of this trial is to evaluate the role of PTR in colorectal cancer patients with asymptomatic, synchronous, unresectable metastasis.

## Methods/design

### Objectives and endpoints

The authors will compare overall survival rates as the primary endpoint measure between a group undergoing PTR followed by chemotherapy and a group undergoing only chemotherapy. Primary tumor-related complication rates, PTR-related complication rates, conversion rates to the resectable state, total cost, and quality of life will be evaluated as secondary endpoint measures. These evaluations will allow us to know whether or not PTR has benefits compared with the upfront chemotherapy strategy in Stage IV colorectal cancer patients with asymptomatic, synchronous, unresectable metastasis.

The primary endpoint measure is a comparison of 2-year overall survival rates between both groups. Secondary endpoint measures are comparisons of quality of life, total cost, and conversion to resectable status between both groups. Moreover, primary tumor-related complication will be evaluated in the group receiving upfront chemotherapy only and PTR-related morbidity and mortality will be evaluated in the group receiving PTR followed by chemotherapy.

### Definition and evaluation methods of parameters

Major primary tumor-related complications are intestinal obstruction, lower gastrointestinal bleeding, perforation of tumor, and fistula. Intestinal obstruction is defined from such symptoms as abdominal discomfort, pain, nausea, vomiting, abdominal distension or tenderness, rebound tenderness on physical examination caused by primary tumor, with evidence of obstruction shown in an abdominal X-radiograph or computed tomograph. Lower gastrointestinal bleeding is defined as a bleeding from the primary tumor, resulting in a reduction in hemoglobin concentration, and requiring transfusion or intervention. Tumor perforation includes both tumor perforation and other site perforation owing to high pressure caused by primary tumor obstruction. Fistula is defined as fistula formation between the primary tumor and an adjacent organ, as observed on physical examination or in imaging studies.

We consider the total cost to be all the colorectal cancer-related cost. It is related not only with the treatment itself, including surgery and chemotherapy, as well as care during admission and in the outpatient department, but also readmission due to adverse events or complications. In Korea, data on the cost of caring for Korean patients can be obtained from the Ministry of Health and Welfare, with approval.

To compare the quality of life between the two groups, the Korean version of EQ-5D^TM^ (EuroQol Group) and the European Organization for Research and Treatment of Cancer Quality of Life Questionnaire-C30 will be used. All the enrolled patients will complete the questionnaires before chemotherapy or PTR, and thereafter every 6 months.

Morbidity and mortality related to PTR are graded according to the Clavien–Dindo classification within 30 days after PTR [21], and chemotherapy-related toxicity is evaluated by the National Cancer Institute Common Terminology Criteria for Adverse Events, version 4.0 [22].

When the state of a tumor converts to resectable during the trial, the treatment plan can be changed into curative intent. Curative or radical resection is defined as complete resection (R0) of both the primary tumor and metastatic lesions, including radiofrequency ablation for liver metastases.

### Study design and period

This is a randomized, prospective, multicenter trial comparing chemotherapy following PTR with chemotherapy alone in colorectal cancer patients with asymptomatic, synchronous, unresectable metastases. The trial has been registered at www.clinicaltrials.gov (NCT01978249).

**Fig. 1:**
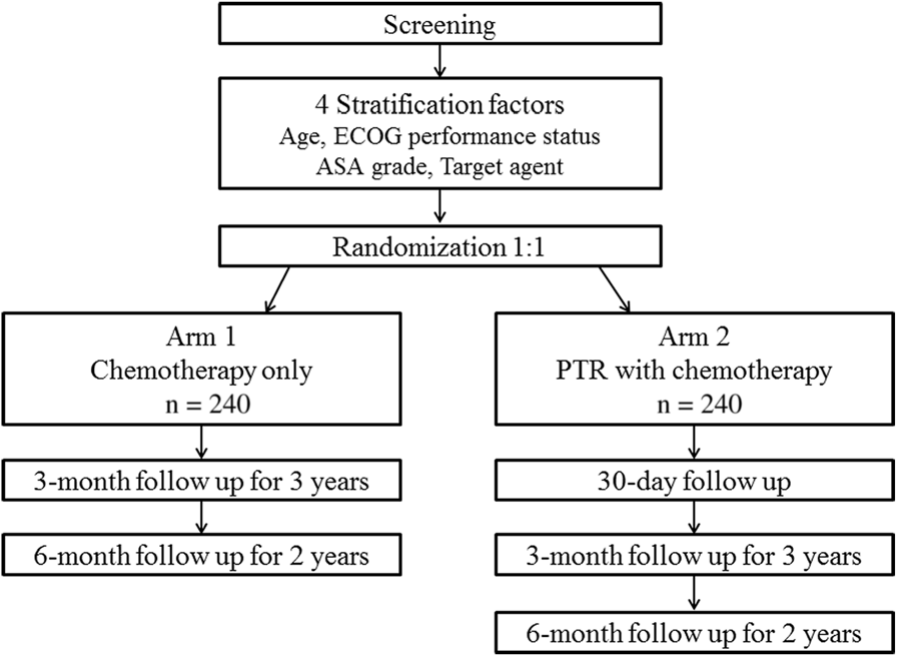
Flow diagram of the trial. ASA, American Society of Anesthesiologists; ECOG, Eastern Cooperative Oncology Group; PTR, primary tumor resection

All patients are randomized in a 1:1 ratio into one of two arms after screening: chemotherapy only (control arm: Arm 1) or chemotherapy after PTR (experimental arm: Arm 2) (Fig. 1). The trial is expected to last 5 years. All eligible patients will be enrolled after approval has been obtained from the institutional review board at each participating institution and enrollment will continue to February 2017. Ethical approval has been received for the trial from the following institutions: Severance Hospital and Gangnam Severance Hospital, Yonsei University College of Medicine, Kyungpook National University Hospital, Soon Chun Hyang University Hospital, Ajou University Hospital, Seoul National University Bundang Hospital, Chonnam National University Hospital, Hallym University Sacred Heart Hospital, National Cancer Center, Yeungnam University Hospital. The accrual period is 3 years and the follow-up period is 2 years.

### Details of treatment method in both comparative groups

**Fig. 2:**
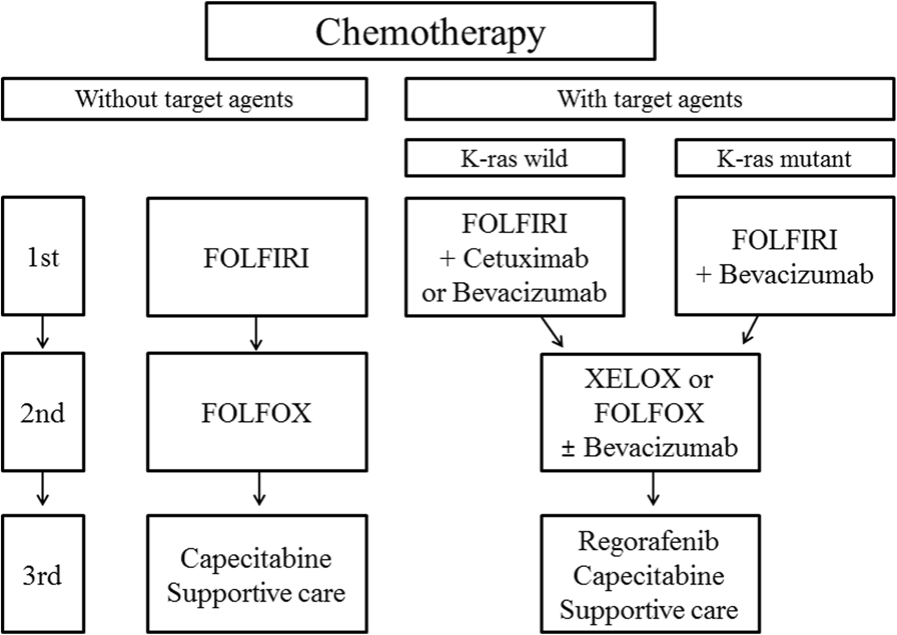
Chemotherapy regimens. FOLFIRI, 5-fluorouracil-based combination therapy with irinotecan; FOLFOX, 5-fluorouracil with oxaliplatin; XELOX, xeloda with oxaliplatin

Patients allocated to Arm 1 (chemotherapy only) will receive chemotherapy first without PTR within 14 days after randomization. They are divided into two chemotherapy groups: chemotherapy with or without target agents. The chemotherapy regimens are outlined in Fig. 2. The use of target agent (cetuximab, panitumumab, bevacizumab) is decided by the physician. This is one of the stratification factors.

If it is decided that the patient will not receive target agent, the first line of chemotherapy is 5-fluorouracil-based combination therapy with irinotecan (FOLFIRI), and the second line is 5-fluorouracil with oxaliplatin (FOLFOX). If patients who received second-line therapy need third-line therapy, they can be prescribed capecitabine or other chemotherapeutic agents or supportive care.

All chemotherapeutic agents will be used according to the guidelines of the national health insurance service of Korea. According to the results of K-ras mutation tests, an appropriate target agent will be used. If the patient wants to receive target agent, the first line of chemotherapy is FOLFIRI with cetuximab or bevacizumab for patients without the K-ras mutation and FOLFIRI with bevacizumab for patients with the K-ras mutation. The second-line therapy is xeloda with oxaliplatin (XELOX) or FOLFOX with or without bevacizumab. The third-line therapy is regorafenib, capecitabine, or supportive care.

Patients allocated to Arm 2 (chemotherapy after PTR) will receive PTR within 14 days after randomization. Then they will receive chemotherapy within 8 weeks after PTR. The chemotherapy regimen is the same as that of Arm 1.

Primary tumor resection is intended to obtain a complete resection of the primary tumor (R0) with negative resection margins and adequate lymphadenectomy. The type of operation will be determined by the location of the tumor, and may be right hemicolectomy, transverse colectomy, left hemicolectomy, or anterior resection. The surgeon can decide the method of operation, whether open or laparoscopic surgery. For the primary site, a complete resection (R0) or microscopic remnant resection (R1) should be confirmed by pathologists after PTR. Patients with visible incomplete resection (R2) are excluded from this study. Operative and pathologic reports are documented in a case report form. All adverse events occurring within 30 days after PTR are recorded, using the Clavien–Dindo classification of surgical complications [21].

### Sample size calculation and randomization

The literature was searched to find a significant difference in 2-year overall survival rates between Arms 1 and 2. Ruo *et al.* [18] reported a 25 % probability of 2-year overall survival in resected patients compared with 6 % in patients never resected (*P* < 0.001). Ferrand *et al.* [13] reported similar results, of 24 % versus 10 % (*P* = 0.001). Galizia *et al.* [14] reported a difference of 21 percentage points (38 % versus 17 %). All the studies documented prolonged survival in the PTR group, as compared with the chemotherapy only group. Thus, we hypothesized that Arm 2 would show an improvement of 10 percentage points (approximately 47 % improvement) in 2-year overall survival rate than Arm 1 (31 % versus 21 %). Considering the size of each center, about 10,000 colorectal patients visit the trial centers yearly. We assumed that 20 % of them would have Stage IV cancer, and 80 % of these would have an unresectable metastasis. Finally, 10 % of patients would not present with any symptom; therefore, approximately 160 patients would be enrolled per year. The accrual period amounts to 3 years and the follow-up period amounts to 2 years. Based on the inequality design, a two-sided log-rank test was conducted, giving an α-error of 0.05 and a power of 80 %, using PASS version 12 (NCSS statistical software, Kaysville, UT, USA). The drop-out rate is expected to be 10 %; therefore, 480 patients (240 per group) must be enrolled.

Of the 480 subjects, 300 will be assigned to Arm 1 or Arm 2 by permuted block randomization within strata with block size 2 or 4. Randomization will be performed in a 1:1 ratio, and stratified according to age (<70 versus ≥70), Eastern Cooperative Oncology Group performance status (0 versus 1 and 2), American Society of Anesthesiologists score (1 and 2 versus 3), and target agent (use versus no use). Because there will not be so many enrolled patients in each center, owing to the rarity of this kind of cancer, we will analyze data first without considering the site effect, and then we will analyze data considering the site effect as a random effect. The remaining 180 subjects will be enrolled using the Pocock and Simon minimization algorithm, to balance the study groups [23]. Although the first 300 patients will be randomized under the stratified randomization, the enrollment speed will vary. This may result in an imbalance between the two groups, so we will use the minimization method for later patients to balance the randomization more precisely.

### Enrollment criteria and detailed definitions

#### Inclusion criteria

1. Aged 20 years old or older
2. Histologically confirmed adenocarcinoma of the colon or upper rectum
3. Resectable primary colon or upper rectal cancer and unresectable metastatic lesions
4. No primary tumor-related symptoms
5. Eastern Cooperative Oncology Group performance status of 0–2
6. Appropriate organ functions (hepatic transaminases – less than five times the normal range; total bilirubin – less than twice the normal range; serum creatinine – less than 1.5 times the normal range; platelets – more than 100,000/μl; neutrophil – more than 1,500/μl)
7. American Society of Anesthesiologists score of ≤ 3
8. Able to give informed consent.

#### Exclusion criteria

1. The patient received adjuvant chemotherapy within the past 6 months
2. The patient received chemotherapy for metastatic colorectal cancer
3. The patient was planning to have curative surgery for the metastatic lesions
4. The primary cancer is unresectable
5. Patients with peritoneal carcinomatosis
6. Patients with mid and low rectal cancer (≤10 cm)
7. Patients with primary tumor-related complications, such as intestinal obstruction, intractable bleeding, and perforation; these need to be treated
8. American Society of Anesthesiologists score of ≥ 4
9. The patient has chronic hepatitis or cirrhosis; an asymptomatic carrier of hepatitis B virus or hepatitis C virus may participate
10. Patients with an active infection, which requires antibiotic therapy, during the randomization period
11. Pregnant or breastfeeding women
12. Patients who are enrolled in another clinical trial during the time of enrollment (within the 28-day randomization period)
13. Patients who have had another different malignant tumor in the previous 5 years; patients with treated non-melanoma skin cancer or cervical cancer may be enrolled.

‘Asymptomatic’ is defined as lack of primary-tumor-related symptoms, including obstruction, lower gastrointestinal bleeding, and perforation. Symptoms of obstruction such as abdominal discomfort, pain, nausea, vomiting must have not developed. There is no abdominal distension or tenderness; rebound tenderness on physical examination should be reported. No evidence of obstruction from abdominal X-ray or computed tomography scan is required. Bleeding from cancer, which results in a reduction in hemoglobin concentration and requires transfusion or intervention, is considered to be ‘symptomatic’. Patients with non-specific symptoms, such as anorexia, dyspepsia, general weakness, bowel habit change, or intermittent hematochezia may be included in this trial.

‘Unresectable primary tumor’ is defined as a tumor in which there is extensive involvement of the vena cava, superior mesenteric artery, pancreas, and duodenum, as observed in preoperative imaging study.

‘Unresectable metastasis’ is defined as follows:

1. After liver resection, the adjacent two segments of liver cannot be preserved, or vascular inflow, outflow, biliary drainage, and pedicle cannot be preserved, or the remnant parenchyma of the liver is expected to be less than 30 % of the total volume, or all the metastatic lesions cannot be resected completely.
2. There are more than five metastatic lesions in the lung parenchyma, or larger than lobectomy is required, even if there are fewer than five metastatic lesions, or the patient has insufficient respiratory function (e.g., forced expiratory volume less than 1 liter per 1 second or less than 60 % of normal level).
3. The metastasis is discovered in the brain, bone, neck, mediastinum, or retroperitoneal area.

### Trial implementation

Informed consent will be obtained from all participants. Patients who have agreed and signed an informed consent form are randomized in a 1:1 ratio by stratification after screening. The first visit is for screening. The participant’s record includes name, screening number, birth date, and date of signature, with the name of institution, date of screening, and eligibility. After obtaining informed consent from a potential patient, an investigator screens the patient with a screening number. Randomization is performed using a computer-generated random sequence. After randomization, treatment begins within 2 weeks.

Evaluation items for screening are:

1. Past medical and surgical history
2. Physical examination
3. Eastern Cooperative Oncology Group performance status
4. American Society of Anesthesiologists score
5. Electrocardiography
6. Complete differential blood count
7. Routine biochemical analysis, including glucose, blood urea nitrogen, creatinine, electrolytes, total protein, albumin, aspartate aminotransferase, alanine aminotransferase, alkaline phosphatase, total bilirubin, and uric acid
8. Tumor marker: carcinoembryonic antigen
9. Urine analysis
10. Colonoscopy and biopsy
11. Blood or urinary β-hCG (in a fertile woman)
12. Chest X-ray
13. Chest computed tomography (not mandatory if the patient has undergone positron emission tomography)
14. Abdominal-pelvic computed tomography
15. Liver, brain, rectum magnetic resonance imaging, bone scan, etc., for diagnosis of metastatic lesion (if indicated)
16. Pulmonary function test for Arm 2 (PTR group) or patient with lung metastasis.

### Follow up

In Arm 1, chemotherapy must start within 2 weeks after randomization. Follow-up appointments for study will take place every 3 months for 3 years after the start of chemotherapy, then every 6 months for 2 years. In Arm 2, PTR must start within 2 weeks after randomization. The next visit will be performed within 30 days after PTR, and chemotherapy must start within 8 weeks after PTR. Follow-up appointments for study are planned every 3 months for 3 years after starting day of chemotherapy, then every 6 months for 2 years.

### Data collection and management

**Fig. 3:**
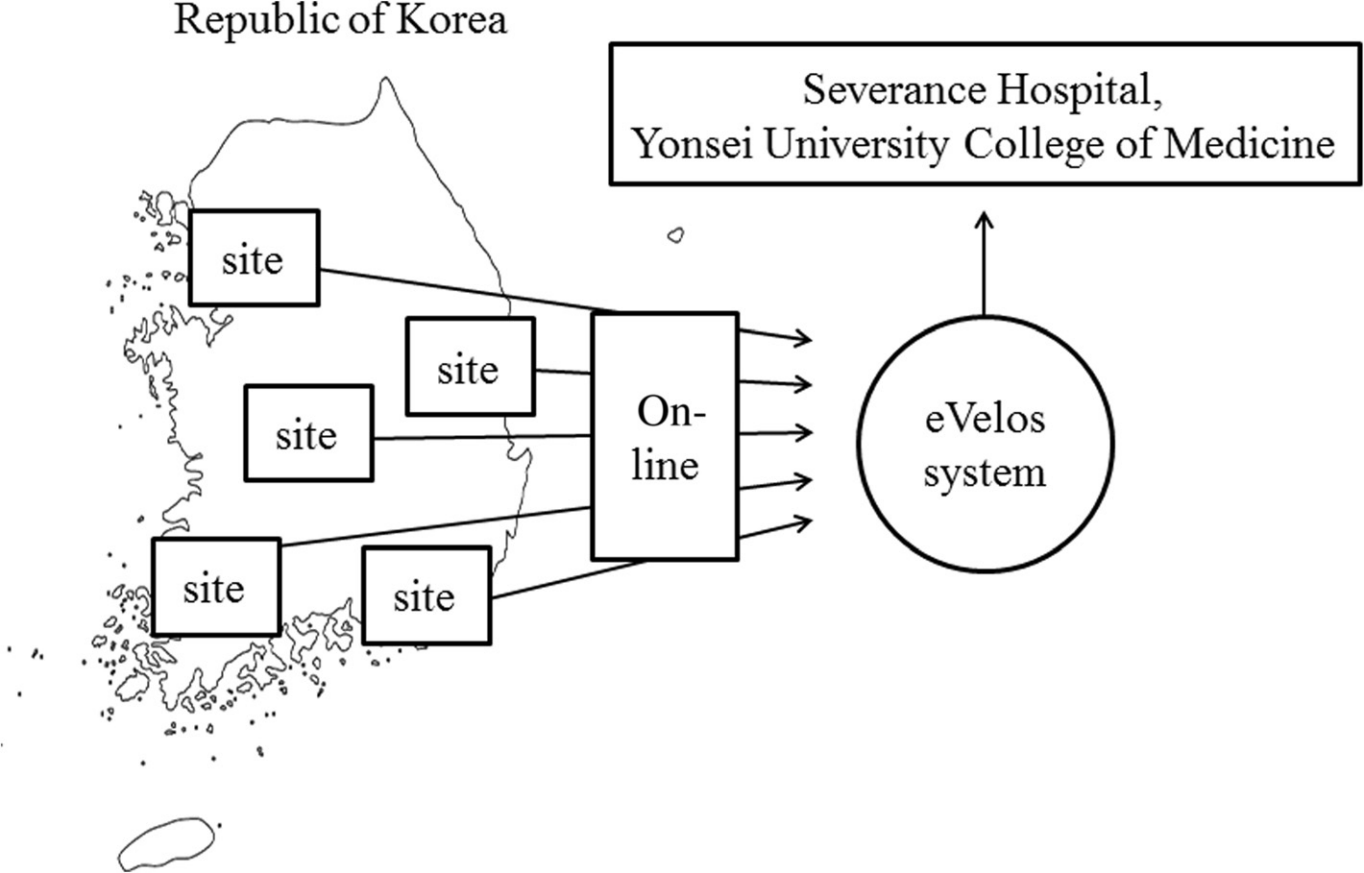
Data collection and management

We invited more than 20 centers having more than 500 beds and performing more than 100 surgeries for colorectal cancer yearly in Korea; 16 of these centers agreed to participate in the trial. Because the sites are located far apart from each other, all clinical data and information should be recorded and sent to our institution. We will employ a web-based clinical research management system (eVelos, provided by National Cancer Center, Ilsan, Republic of Korea), which gathers clinical data and information on-line. Principal investigators or clinical research coordinators in each site should sign up to the eVelos system and be trained in its use, and they will enter clinical data onto an electronic case report form. Approved researchers in our institution will manage the data and information (Fig. 3).

### Dropping out

A participant can drop out of the study in the following cases:

1. Unacceptable adverse events
2. An investigator stops for a participant’s benefit
3. Different treatment is required, which is not approved in this trial
4. A participant refuses for any reason
5. A participant becomes pregnant, or discovers that she is pregnant
6. Poor compliance

### Statistical analysis

All patients who were allocated to either Arm 1 or Arm 2 by randomization will be included and their data will be analyzed. Categorical and continuous data of the patients for each intervention group will be analyzed. Two-year overall survival rate, the primary endpoint measure of this trial, will be estimated using Kaplan–Meier methods. In addition, the hazard ratio will be compared by Stratified Cox’s proportional hazards model. The assumption of proportional hazards for the two study groups will be checked by log-minus-log survival plots and the time-dependent covariate test.

Secondary endpoint measures will be analyzed as follows: primary tumor-related complication will be evaluated in the group receiving chemotherapy only and PTR-related morbidity will be evaluated in the group receiving PTR followed by chemotherapy. The quality of life, as assessed using the aforementioned questionnaires, will be compared between both arms using a linear mixed model. The total cost until closure of trial will be compared using a *t* test in both arms. Significance will be considered for *P* less than 0.05. All analyses will be performed using SPSS version 20.0 (SPSS Inc., IBM Corp., Armonk, NY, USA), SAS (version 9.2, SAS Inc., Cary, NC, USA), and R package, version 3.0.3 (https://www.R-project.org).

### Safe evaluation and reporting of adverse effect

Adverse events and serious adverse events must be reported to protect participants. Complications, including surgery-related morbidity, primary tumor-related complications, and chemotherapy-related toxicity, should be recorded in the electronic case report form. Serious adverse events should be reported every 3 months; suspected, unexpected, serious, adverse reactions, which could result in death or are life threatening, should be reported within 15 days of detection by investigators.

### Data monitoring

A committee will be organized for trial supervision. All members of the committee are certified according to the course of good clinical practice in each institution. All process including data collection, records, and management will be monitored by them. The committee will check the interim results at least yearly, and they can advise and consult the principal investigator. Interim results will also be reported to the Ministry of Health and Welfare yearly for the overall trial period. If the interim results are considered not to sustain the trial (i.e., significant better or worse treatment arm, excessive morbidity), the chief of the Ministry of Health and Welfare can decide to withdraw an institution or cancel the entire trial.

### Ethical and legal considerations

The authors follow the Declaration of Helsinki to protect the patients, and the trial will be performed according to the guidelines of the International Council for Harmonization – Good Clinical Practice. Each institution that participates in this trial must obtain approval from its own institutional review board. All patients will understand and agree to the aims and process of the trial, and the possible results and risks. An informed consent form must be written in language that the patients can understand, and must be explained by an investigator. If patients cannot read an informed consent form, an investigator must read it in the presence of a witness. Although a patient has signed an informed consent form at first, an investigator must cancel the process if the patient subsequently refuses. A copy of the signed informed consent form should be provided to the patient. An original copy will be held in an investigator’s safe keeping.

Medical records of the patients can be reviewed for the aims of the trial only by strictly authorized persons. Randomization number and initials of patients are recorded in the case report form, but not other data, such as full name or registration number.

## Discussion

Between PTR with chemotherapy and chemotherapy only, which is better has remained unclear and controversial for the treatment of asymptomatic colorectal cancer patients with unresectable metastasis considering oncologic outcomes. Moreover, in terms of complications, Poultsides *et al.* [11] reported that 11 % of 233 patients with synchronous Stage IV colorectal cancer developed primary tumor-related complications and only 7 % required emergent surgery. In their study, seven patients underwent intraluminal stenting successfully. Eventually, Poultsides *et al.* [11] concluded that their data supported the use of chemotherapy without routine prophylactic resection, as the appropriate standard practice for patients with neither obstructing nor hemorrhaging primary colorectal tumors in the setting of metastatic disease. Meanwhile, in our data, primary tumor-related complication rates of 252 patients in our institution were 35 %, and 8 % required emergent surgery [20]. Moreover, Van Hooft *et al.* [24] closed their multicenter randomized clinical trial comparing endoscopic stenting versus surgery for Stage IV left-sided colorectal cancer because of high numbers of complications (i.e., 6 perforations of 11 patients who underwent stent insertion). They reported that the safety of non-operative intervention instead of PTR was questionable.

Postoperative mortality rates following palliative resection were reported to be 8.3–9.8 % by publications before 2000 [17, 25, 26]. However, these rates have decreased to 0–5 % since 2000 [12, 14, 18]. This decrease would be because of development of operative technique, anesthesiology, and postoperative management. According to data from our institution, the 30-day mortality rate after PTR was 1.9 % [20]. These results favor PTR.

However, all these retrospective studies cannot be used to establish treatment guideline because of their inherent bias. Thus, this trial is proposed and aims to provide critical data, to support the establishment of a standard treatment protocol for patients with asymptomatic Stage IV colorectal cancer with unresectable metastasis. In this clinical trial, quality of life is secondary endpoint measure. If this clinical trial finds any differences of quality of life between two treatment groups, this will be invaluable knowledge for the patients even if oncologic outcomes are similar between treatment methods. Another secondary endpoint measurement is comparison of hospital cost between the two treatment methods. This comparison will provide important knowledge not only for patients but also for government health policy makers, who want to establish a cost-effective medical system.

The trial started with the first enrolled patient in October 2013. The authors expect that this trial will find an optimal treatment strategy for asymptomatic colorectal cancer patients with unresectable metastasis with level 1 evidence.

## Trial status

This study is currently in the recruitment phase.

## Abbreviations

FOLFIRI: 5-fluorouracil with irinotecan
FOLFOX: 5-fluorouracil with oxaliplatin
PTR: primary tumor resection
SAS: statistical analysis aystem
SPSS: statistical package for the social sciences
XELOX: xeloda with oxaliplatin
